# Swedish Medical Gymnastics in Chronic Diseases of the Heart

**Published:** 1900-06

**Authors:** E. Christofferson

**Affiliations:** Royal Central Institute of Gymnastics, Stockholm


### SWEDISH MEDICAL GYMNASTICS IN CHRONIC DISEASES OF THE HEART.

BY

E. CHRISTOFFERSON

*(Royal Central Institute of Gymnastics, Stockholm).*

---

EVER since the introduction of Swedish Medical Gymnastics by Per Henrik Ling at the beginning of the 19th century, this method of treatment has been applied with great success in certain diseases of the heart. Thus in many forms of chronic heart affections the circulation is necessarily weakened, and the object of medical gymnastics will therefore be *to facilitate the work of the heart* by improving the weakened circulation, and further *to strengthen the heart itself* by active exercises in cases where the use of such exercises is indicated.

In our medical gymnastics we have three groups of passive exercises, which all have a great effect in furthering the circulation; viz., kneadings, rollings, and respiratory movements.

*Muscle-kneading* is a kind of massage, although for the sake of convenience called a passive exercise. It has the effect of squeezing out the blood, so to speak, from the muscles and the intramuscular veins in the direction towards the heart. It acts in this way, as von Mosengeil aptly expresses it, simultaneously as a pressure- and a suction-pump. It increases the lumen of the peripheral vessels, and thus relieves the heart from part of its load. *Abdominal-kneading* is given, even in the absence of disorders of the digestion, on account of its circulatory-furthering effects, especially in some valvular lesions with venous congestion in the digestive organs. The circulatory-furthering effect of the abdominal-kneading is increased by the fact that it causes a mechanical excitation of the splanchnic nerves, and through these a contraction of the mesenteric arteries as well as of the venæ portæ and the vena cava inferior.

The second group are the *rollings*, in the ankle, hip, shoulder, and wrist joints. It is rather difficult to give a short and intelligible description of these movements. They consist of the combined movements of extension, abduction, flexion, and adduction in the respective joints so as to produce a rolling, performed by the operator, while the patient is quite passive, in sitting or recumbent position. The effect of the successive flexion and extension will be a lengthening and shortening of the veins near the joints, and thus a suction of blood from the nearest peripheral parts. These movements are especially effective in the shoulder and hip joints on account of the big veins here and the fasciæ, which are attached to them and help to make them taut. The effect of this kind of movement is apparent by the warmth experienced in the feet after a foot-rolling. Trunk-rolling is almost always used in the treatment of heart diseases.

Of perhaps still more importance than the exercises of the two previous groups, which mainly influence the peripheral circulation, are the *respiratory movements.* They are passive exercises for making the respiration deeper. As is well known, the consequence of a deeper inspiration is a stronger suction of blood to the right heart and also an increased pulmonary circulation. As an incomplete oxidation of the blood follows a weakened circulation, it is clear that also from this point breathing exercises are of importance in heart diseases.

Besides the above-mentioned exercises for improving the circulation we have another group of passive exercises, which have a soothing effect on the action of the heart. Of these is especially to be mentioned the so-called "local heart-treatment." This consists of light strokings over the heart and in a light "tremble-shaking," performed with one of the operator's hands placed over the heart of the patient. The explanation of the influence of these manipulations may be a reflex action on the vagus nerve, although the possibility of a psychical influence cannot be excluded. According to researches made by Dr. Levin, of Stockholm, in 6000 observations during a period of ten years, this local heart-treatment has the power of diminishing the pulse rate by 8—12 beats a minute. The above-mentioned abdominal-kneading has the same effect, in a somewhat lesser degree.

The effect of the passive exercises will thus be a more normal distribution of the blood mass. The venous congestion is diminished by the increased flow of blood to the right heart and by the fact that the heart can more easily pump away the blood. The blood is drawn more to the peripheral parts and the arterial circulation is increased by the diminished peripheral resistance. The overburdened heart, relieved from part of its load, is able to contract more fully and slowly, and will gain strength from this and from an improved coronary circulation.

The passive exercises all tend to improve the circulation and relieve the heart. But our aim will also be, in certain cases, to strengthen the heart-muscle by *gradually increasing its work.* This is done by *active exercises.* These exercises are practically the same as those used in the "Nauheim treatment," with the exception however of those in which the arms are carried above the level of the shoulders. The exercises are performed by the patient, as a rule either in a sitting or recumbent position, slowly and under full breathing, while the operator makes a moderate resistance. As a rule we begin with exercises for the legs, as these are found to be better tolerated by the patients than the arm exercises, probably because the latter tend to make the respiration more difficult. This fact, too, agrees well with the principles and good effects of the "Oertel terrain-cure," in which active exercises for the lower extremities only are used.

The influence of the active exercises is, to a certain extent, the same as that of the passive; they unload the heart by drawing the blood to the working muscles. Thus the heart can more easily do the increased work demanded of it, contracts more slowly and completely, and grows gradually stronger at the same time as the other working muscles.

But however slowly and cautiously the active exercises may be done, they always increase the work of the heart, and are not given therefore in more severe cases with failing compensation. It will here only be a matter of helping the circulation and relieving the heart without increasing its work. If the symptoms of failing compensation have thus been relieved, we may gradually add active exercises.

It is evident that the time given to the treatment, as well as the strength and number of active exercises, differ according to the case treated and the condition of the patient. As space does not allow me to go into details in the treatment of different kinds of heart diseases, it may briefly be said that passive exercises are given in all forms of heart disease treated by gymnastics, that is in cases of valvular affections, cardiosclerosis and chronic myocarditis, fatty infiltration, idiopathic hypertrophy and dilatation of the heart ("weakened heart"). Also in some heart neuroses (nervous palpitation, morbus Basedowi and others) the gymnastic treatment has shown itself valuable.[1]

The active exercises are especially used in cases of fatty infiltration, some heart neuroses, and in valvular lesions with fairly good compensation. They *may* in other cases, according to what has been said above, subsequently be added to the passive exercises. As a rule they are not given in cases associated with aneurysma aortæ or advanced arterio-sclerosis. The exercises given in the following case may serve as an example of a "gymnastic prescription":—

F. F. C., railway engineer, was referred to me by Dr. Watson Williams, complaining of palpitation, a sense of oppression in the chest and an irritable cough with slight mucous expectoration. The first cardiac sound at the apex was prolonged and there was a marked epigastric pulsation with an indefinite bruit over the tricuspid area. The symptoms dated from excessive muscular exertion, and were partly attributable to excessive smoking, but he had been very much overworked and had had a great deal of worry in connection with his occupation. Under his physician's supervision I gave him the following treatment:—

1. Sitting chest-lifting (a passive respiratory exercise).
2. Foot-rolling, with active foot flexion and extension.
3. Leg-muscle-kneading.
4. Leg-rolling (in the hip joint), with active flexion and extension in the hip and knee joints respectively.
5. Arm-muscle-kneading, rolling in the shoulder joints and active flexion and extension of the forearm.
6. Kneading of the back muscles, back hacking and nerve friction (as a stimulant).
7. Trunk-rolling.
8. Abdominal kneading.
9. Local heart-treatment.
10. Sitting chest-lifting.

[1] Wide, *Handbook of Medical Gymnastics.*

After every second or third exercises three or four chest-liftings and a few minutes' rest were given. The treatment was continued for an hour daily for four weeks. The result was, according to Dr. Watson Williams, that "the heart became much quieter, the heart's action became normal and the general health was immensely improved."

Dr. Wide, the Head Physician of the Gymnastic Orthopedic Institute, Stockholm, in his *Handbook of Medical Gymnastics*, says :—

"Even if medical gymnastic treatment can in no case free the patient from the heart disease itself and only in few cases improve it, still I dare assert, on account of the experience I have gained during the course of years, that every patient with heart disease will obtain relief from the troublesome symptoms of palpitation, shortness of breath, pain and oppression over the heart, with other symptoms that always accompany heart disease—and even this is much to be thankful for.

"It is true that the improvement will not be lasting in every case, but that the treatment must be often repeated and for long periods; but it should also be remembered that the disease is chronic, and that no other treatment gives better result. I know several heart patients who year by year continue to take medical gymnastics and just on this account are able to keep up and continue to be hard-working people, when otherwise they would be condemned to an inactive life, often, perhaps, to a constant sick-bed.

"Finally, it should perhaps be pointed out, that medical gymnastics, as well when it is a question of treating heart disease as any other illness, does not exclude a simultaneous use of medicinal treatment, and that it is often necessary to employ both in order that the patient may obtain the greatest possible benefit."

